# Molecular mechanisms underlying cyclophosphamide-induced cognitive impairment and strategies for neuroprotection in preclinical models

**DOI:** 10.1007/s11010-023-04805-0

**Published:** 2023-07-31

**Authors:** Kamilia M. Ibrahim, Samar F. Darwish, Eman M. Mantawy, Ebtehal El-demerdash

**Affiliations:** 1 Department of Pharmacology & Toxicology, Faculty of Pharmacy, Ain Shams University, Cairo, Egypt; 2 Department of Pharmacology & Toxicology, Faculty of Pharmacy, Badr University in Cairo (BUC), Badr City, Cairo 11829 Egypt; 3 Preclinical and Translational Research Center, Faculty of Pharmacy, Ain Shams University, Abasia, Cairo Egypt

**Keywords:** Cyclophosphamide, Neurotoxicity, Molecular mechanisms, Protective strategies

## Abstract

Cyclophosphamide has drastically enhanced the expectancy and quality of life of cancer patients. However, it is accompanied by diverse neurological complications which are considered a dose-limiting adverse effect. Neurotoxicity caused by cyclophosphamide can manifest in numerous manners including anxiety, depression, motor dysfunction and cognitive deficits. This review article offers an overview on cyclophosphamide-induced neurotoxicity, providing a unified point of view on the possible underlying molecular mechanisms including oxidative brain damage, neuroinflammation, apoptotic neuronal cell death as well as disruption of the balance of brain neurotransmitters and neurotrophic factors. Besides, this review sheds light on the promising protective agents that have been investigated using preclinical animal models as well as their biological targets and protection mechanisms. Despite promising results in experimental models, none of these agents has been studied in clinical trials. Thus, there is lack of evidence to advocate the use of any neuroprotective agent in the clinical setting. Furthermore, none of the protective agents has been evaluated for its effect on the anticancer activity of cyclophosphamide in tumor-bearing animals. Therefore, there is a great necessity for adequate well-designed clinical studies for evaluation of the therapeutic values of these candidates. Conclusively, this review summarizes the molecular mechanisms accounting for cyclophosphamide-induced neurotoxicity together with the potential protective strategies seeking for downgrading this neurological complication, thus enhancing the quality of life and well-being of cancer patients treated with cyclophosphamide.

**Graphical abstract:**

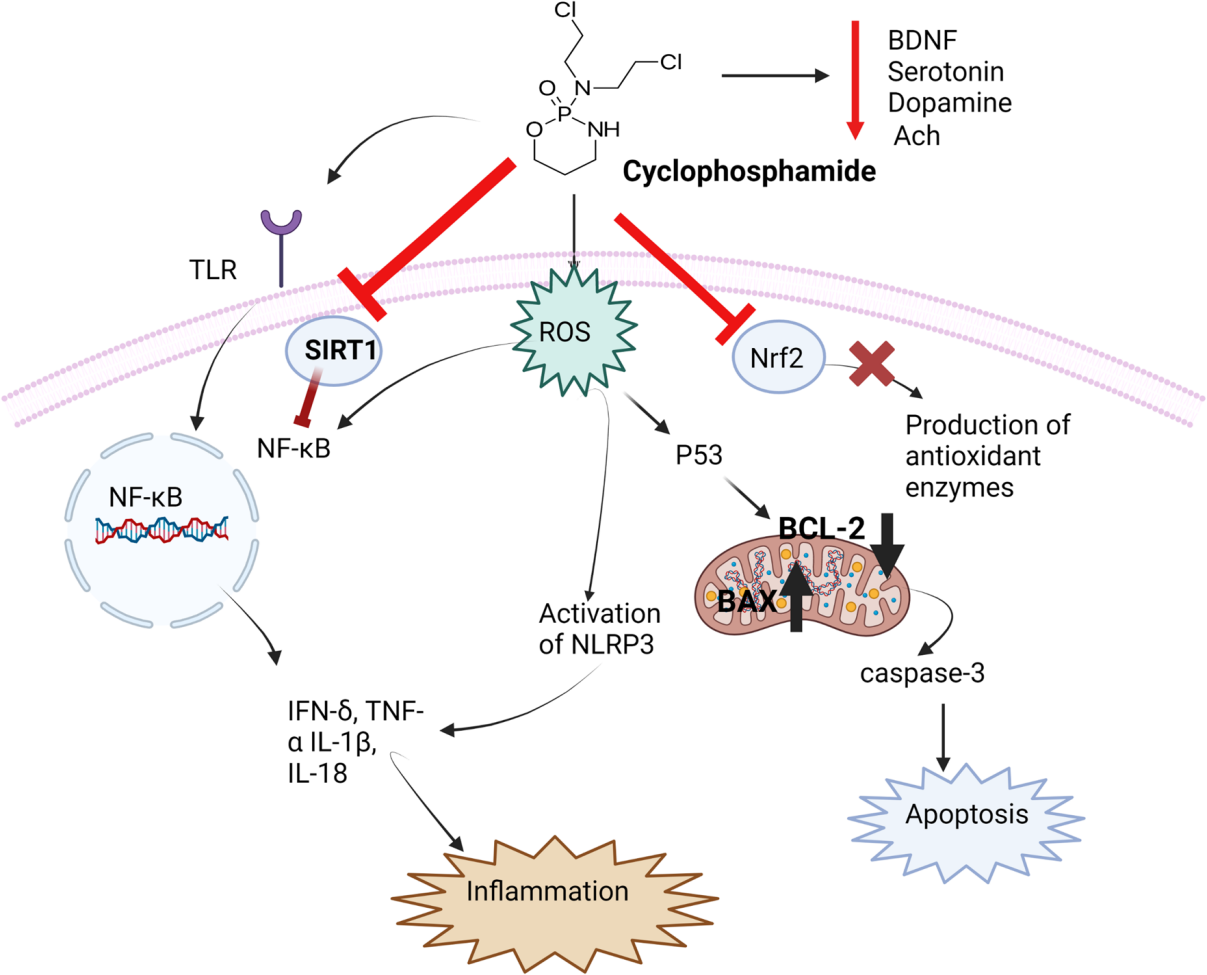

## Literature search

Online research was carried out using medical research databases Google Scholar, NCBI, and PUBMED with the following terms: cyclophosphamide, cyclophosphamide pharmacokinetics, cyclophosphamide pharmacodynamics, cyclophosphamide neurotoxicity human, cyclophosphamide neurotoxicity animals, cyclophosphamide cognitive impairment, cyclophosphamide oxidative stress, cyclophosphamide inflammation animals, cyclophosphamide apoptosis.

Date of last search: 25th April 2023. Inclusion Criteria: Priority was given to primary original articles with higher empirical values, evidence-based methodology, narrative and systematic reviews in English Language only.

## Introduction

Chemotherapy-induced cognitive impairment (CICI) is a growing term indicating the neurotoxic effect of chemotherapeutic drugs. CICI is characterized by different forms of cognitive impairments, including impairment of learning, memory, and concentration that impact the quality of life [1]. One of the chemotherapeutic drugs that was reported to induce CICI is cyclophosphamide. The clinical reports indicated that the use of cyclophosphamide together with other cytotoxic agents such as doxorubicin, methotrexate and 5-fluoruracil was associated with cognitive impairment, encephalopathy and other neurological complications experienced by cancer patients [2–6]. However, there is no reported clinical trials on neuroprotective strategies in this situation. The aim of this review is to systematically outline the molecular mechanisms underlying cyclophosphamide associated neurotoxicity and the potential protective strategies for diminishing this neurological complication.

Cyclophosphamide is considered as an inactive prodrug which must be activated enzymatically by the CYP450 family isozymes (CYP2B6, CYP2C9, and CYP3A4), yielding two metabolites [7]. The first metabolite is a nitrogen mustard compound known as phosphoramide mustard, and it is considered the therapeutically active metabolite because it possesses substantial DNA-alkylating activity by sliding between the two strands of DNA and damaging it [8]. The other metabolite is acrolein, which is formed during activation of 4-hydroxy cyclophosphamide to generate active phosphoramide. It is a highly reactive metabolite that can accounts for majority of cyclophosphamide-induced organ toxicities by impeding with the body's antioxidant defense through generating reactive oxygen species (ROS) such as superoxide radicals, and hydrogen peroxide, and is considered the toxic moiety responsible for most of adverse effects of cyclophosphamide [9]. Despite the paucity of data concerning its central nervous system (CNS) penetration, it has been reported that both cyclophosphamide and its first metabolite; 4 hydroxy cyclophosphamide can cross blood brain barrier (BBB) [10].

## Molecular mechanisms underlying cyclophosphamide-induced neurotoxicity

Several molecular mechanisms have been implicated in driving the central nervous system changes leading to cognitive decline in CICI, including the ability of cyclophosphamide to induce oxidative stress, increase inflammation, promote apoptosis and disrupt neurotransmitters and neurotrophic factors.

### Induction of oxidative stress

**Fig. 1:**
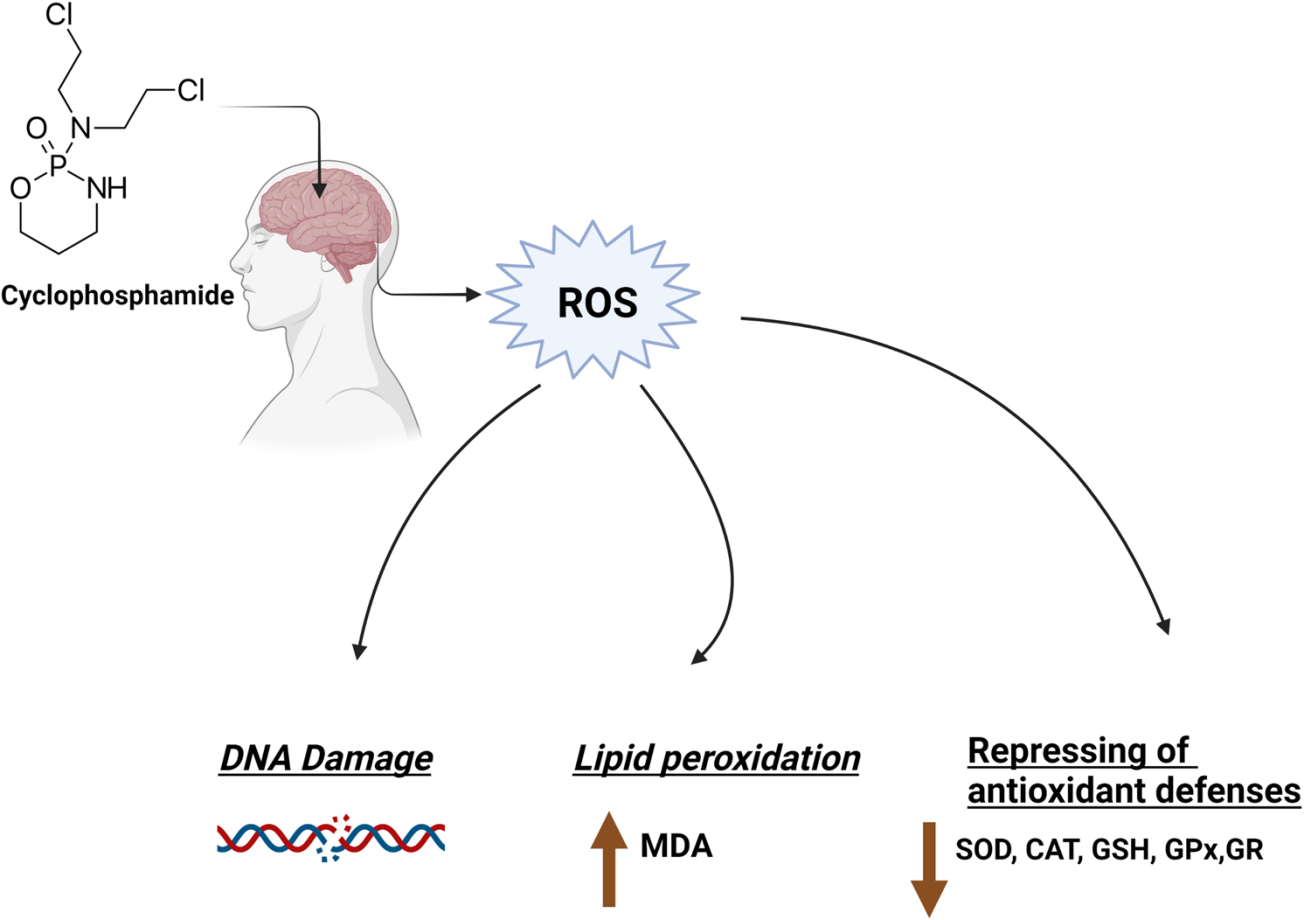
Cyclophosphamide-induced oxidative stress. ROS: Reactive oxygen species, MDA: Malondialdehyde, SOD: Superoxide dismutase, CAT: Catalase, GSH: Glutathione, GPx: Glutathione peroxidase, GR: Glutathione reductase

Oxidative stress is described as the cornerstone for the pathophysiology of cyclophosphamide-induced neurotoxicity. The brain tissues are critically vulnerable to oxidative damage as a result of their limited antioxidant capacity [11]. Neurons and other brain cells are incapable of producing glutathione (GSH), so they rely on the surrounding astrocytes for GSH precursors [12]. In addition, various studies demonstrated that oxidative stress elicited by cyclophosphamide in the brain is mediated through overproduction of reactive oxygen species (ROS), GSH depletion and inhibition of antioxidant enzymes activities [13, 14]. In addition, it was found that the activation of angiotensin converting enzyme and the resultant excessive production of angiotensin II by cyclophosphamide has been implicated in ROS generation and hence the subsequent oxidative brain injury [15].The massive generation of ROS prompts oxidative damage to numerous cellular components resulting in lipid peroxidation, DNA damage and mitochondrial dysfunction [14, 16–18]. Mechanisms by which cyclophosphamide-induced oxidative stress is shown in Fig. 1.

**Fig. 2:**
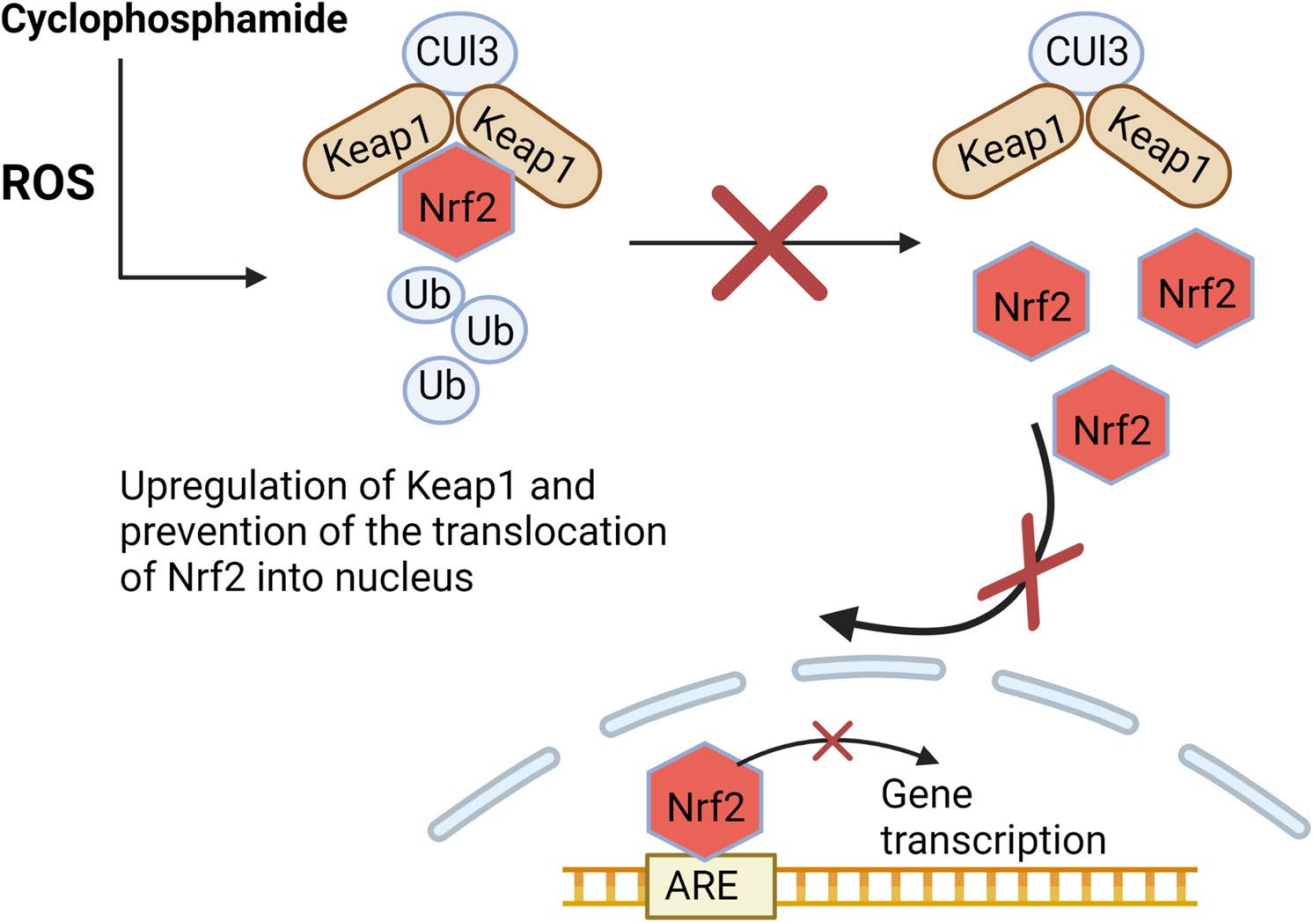
Cyclophosphamide -induced downregulation of Nrf2 pathway. CUl3: Cullin 3, Keap1: Kelch-like ECH-associated protein 1, Nrf2: Nuclear factor erythroid 2-related factor 2

Beside generation of ROS, cyclophosphamide inhibits the endogenous antioxidant pathways, in particular nuclear factor erythroid 2-related factor 2 (Nrf2) signaling pathway, a master transcription factor for conserving redox homeostasis [19]. Under normal circumstances, Nrf2 is sequestered in the cytosol by the action of Kelch-like ECH-associated protein 1 (Keap1) which functions as an adaptor protein for the Cullin 3 (Cul3) containing E3 ubiquitin ligase enzyme. This enzyme is accountable for ubiquitylation and proteasomal degradation of Nrf2 [20]. In contrast, under stressed conditions, certain stress-sensing residues in Keap1 are oxidized, resulting in its conformational changes, inactivation and ultimately its dissociation from Keap1-Nrf2 complex allowing Nrf2 to escape degradation [21]. This consequently results in Nrf2 stabilization, accumulation and translocation into the nucleus where it combines with the corresponding antioxidant-response elements (ARE) switching on the transcription machinery of antioxidant target genes such as heme oxygenase-1 (HO-1), and glutathione reductase (GR) enzymes [22]. Cyclophosphamide downregulated Nrf2 expression and diminished expression of antioxidant enzymes in both hippocampal and cortical tissue [23–25]. Mechanism of cyclophosphamide -induced downregulation of Nrf2 pathway is illustrated in Fig. 2.

### Induction of inflammatory pathway

Ample evidence has verified the crucial role of neuroinflammation in the development and progression of chemotherapy-associated neurological abnormalities [26, 27]. Likewise, several experimental studies have implied that sustained inflammatory responses alongside the immense discharge of pro-inflammatory cytokines as tumor necrosis factor alpha (TNF-α) and interleukins are the major driving force for neurodegeneration associated with cyclophosphamide [17, 23, 24, 28–31]. In this regard, several inflammatory signaling cascades are considered as integral elements in the pathogenesis of neuronal inflammation induced by cyclophosphamide such as nuclear Factor kappa B (NF-κB), toll-like receptors (TLR) and the nucleotide-binding oligomerization domain (NOD)-like receptor family pyrin domain containing 3 (NLRP3) signaling pathways [24, 29, 32].

#### Activation of nuclear factor kappa B (NF-κB) pathway

NF-κB is regarded as the prime transcription factor controlling the expression of a battery of pro-inflammatory genes. It consists of five proteins, p50, p52, p65 (Rel-A), c-Rel, and Rel-B that combine with each other to form distinctive heterodimeric complexes [33]. Under normal conditions, NF-κB complex is inactively maintained in the cytosol through merging with members of its inhibitory proteins called inhibitors of κB (IκB) [34]. Upon activation by various noxious stimuli such as ROS, the IκB kinase (IKK) complex is activated, leading to phosphorylation of IκB, targeting it for ubiquitination and consequent proteasomal degradation [35]. Afterwards, NF-κB becomes unmasked then translocated into the nucleus, where it binds to distinct DNA sequences inducing the transcription of myriad of pro-inflammatory target genes including cytokines, chemokines, enzymes and others [36].

**Fig. 3:**
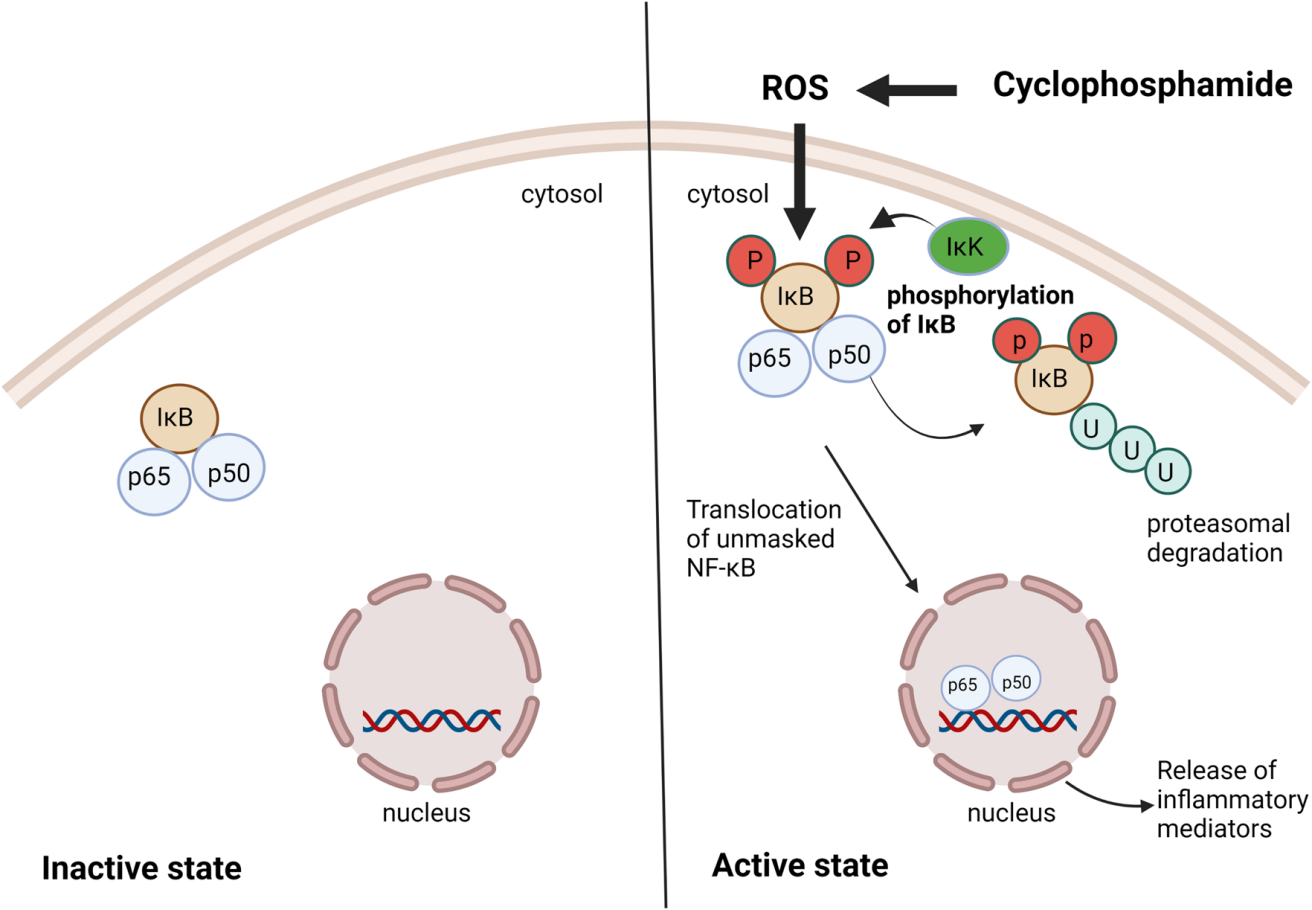
Activation of NF-ΚB pathway by cyclophosphamide. IκB: Inhibitors of κB, IκK: IκB kinase

Notably, abundant studies have confirmed that cyclophosphamide-induced neuronal cell injury is tightly linked with the aggravated inflammatory reactions mediated by the upregulation of NFκB expression along with the subsequent overproduction of inflammatory mediators, including interleukin 1 beta (IL-1β), IL-6, TNF-α, and nitric oxide (NO) [37]. Activation of NF-ΚB pathway by cyclophosphamide is illustrated in Fig. 3.

#### Activation of toll-like receptors (TLRs) pathway

TLRs belongs to a category of transmembrane pattern recognition receptors (PRRs) that are considered as the keystone of the innate immune system [38]. They are expressed on most of innate immune cells such as dendritic cells (DCs), macrophage, and mast cells [39]. Growing evidence has confirmed that TLRs are extensively upregulated in diverse forms of neurological disorders where TLR activation provokes the generation of pro-inflammatory cytokines, resulting in microenvironment damage that is closely linked to neuronal injury [40, 41]. Additionally, the substantial role of TLR activation in the neuroinflammation and behavioral changes associated with use of chemotherapeutic drugs has been proved [42]. In this context, recent studies have affirmed the correlation between cyclophosphamide-induced neurotoxicity with TLR4 activation and the consequent overproduction of pro-inflammatory cytokines and their imperative roles in the pathophysiology of the associated cognitive impairment [32, 43].

The mechanism of signal transduction of TLR follows two major routes; MyD-88 dependent and independent pathways through recruitment of a set of adaptor proteins that further propagate the activation signals amplifying the pro-inflammatory responses [44]. In the MyD88 dependent- pathway, TLR binds with MyD88 at the toll-interleukin 1 receptor (TIR) domain-containing adaptor protein (TIRAP) inducing the recruitment of the IL-1 receptor-associated kinase (IRAK) [45]. Once activated, IRAK provokes activation of TNF receptor-associated factor 6 (TRAF6) [46]. These signaling cascades finally leads to phosphorylating and activating IKK complex which in turn phosphorylates IκB exposing it to the proteasomal degradation, thus unmasking NF-κB, permitting its nuclear translocation and eventually triggering the transcription of an assortment of pro-inflammatory genes [47]. Moreover, TRAF6 can also phosphorylate hence, stimulate mitogen-activated protein kinases (MAPKs), which in turn induce the activation of multiple transcription factors, including activator protein 1 (AP-1) that further induce the expression of several inflammatory mediators [39]

**Fig. 4:**
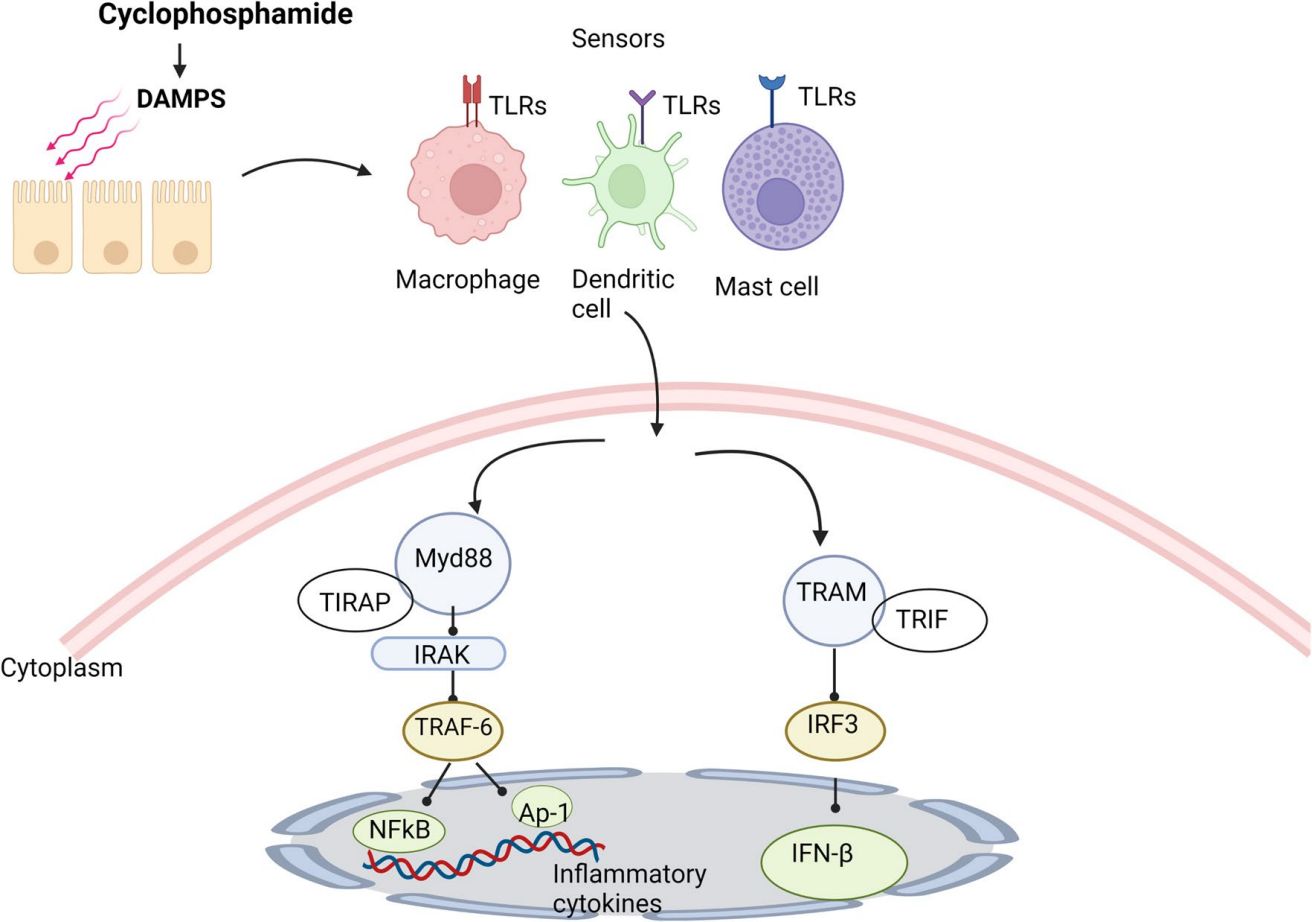
Activation of TLR pathway by cyclophosphamide. TLRs: Toll-like receptors, Myd88: Myeloid differentiation primary response 88, TIRAP: toll-interleukin 1 receptor (TIR) domain-containing adaptor protein, TRAF-6: TNF receptor-associated factor 6, NFkB: Nuclear factor kappa B, Ap-1: activator protein 1, TRAM: Tumor associated macrophage, TRIF: TIR-domain-containing adapter-inducing interferon beta, IRF3: Interferon regulatory factor 3, IFNs: Interferons, IRAK: IL-1 receptor-associated kinase

On the other side, in the MyD88 independent- pathway, TLR recruits the adaptor proteins including TIR-domain-containing adaptor-inducing interferon (TRIF) and translocating chain-associated membrane protein (TRAM) [48]. Upon dimerization of these two proteins, the interferon regulatory factor 3 (IRF3) becomes activated inducing the expression and release of interferon beta (IFN-β) [49]. Figure 4 depicts the Activation of TLR pathway by cyclophosphamide.

#### Activation of NLRP3 inflammasome/caspase 1 signaling pathway

**Fig. 5:**
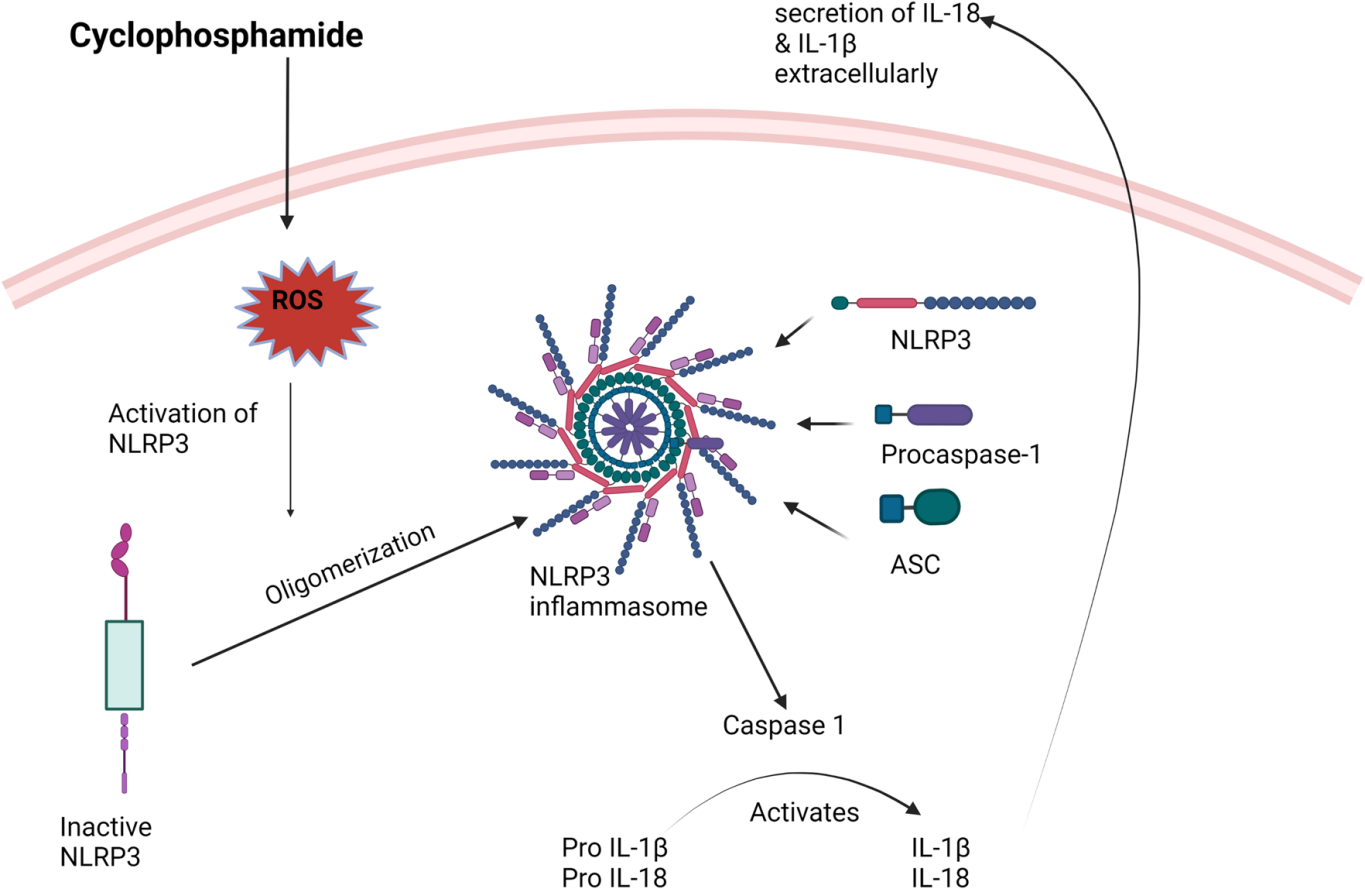
Activation of NLRP3 inflammasome/Caspase 1 signaling pathway by cyclophosphamide. NLRP3: the nucleotide-binding oligomerization domain (NOD)-like receptor family pyrin domain containing 3, IL-1β: Interleukin 1 beta, ASC: Apoptosis-associated speck-like protein

Inflammasomes are a cluster of multiprotein that assemble in the cytoplasm and become activated in response to an assortment of endogenous and exogenous stimuli such as ROS, PAMPs and DAMPs [50]. Multiple subtypes of inflammasomes have been recognized. Among them, NLRP3 inflammasome is the most distinguished one [51]. Upon activation, NLRP3 inflammasome is formed via assembly of NLRP3, apoptosis-associated speck-like protein (ASC), and pro-caspase 1 [50]. Subsequently, this leads to activation of pro-caspase 1 into active caspase 1 which in turn cleaves the cytokine precursors; pro-IL-1β and pro-IL-18 into their mature and active forms; IL-1β and IL-18 and consequently stimulate production of other inflammatory cytokines, and thus exacerbating the inflammatory reactions [52]. Several reports have exhibited the upregulation of NLRP3 inflammasome signaling cascade in the pathogenesis of neuronal inflammation coupled with cyclophosphamide-induced neurodegeneration and behavioral changes [29, 32, 53]. Figure 5 represents the activation of NLRP3 inflammasome/Caspase 1 signaling pathway by cyclophosphamide.

#### Downgrading SIRT-1

**Fig. 6:**
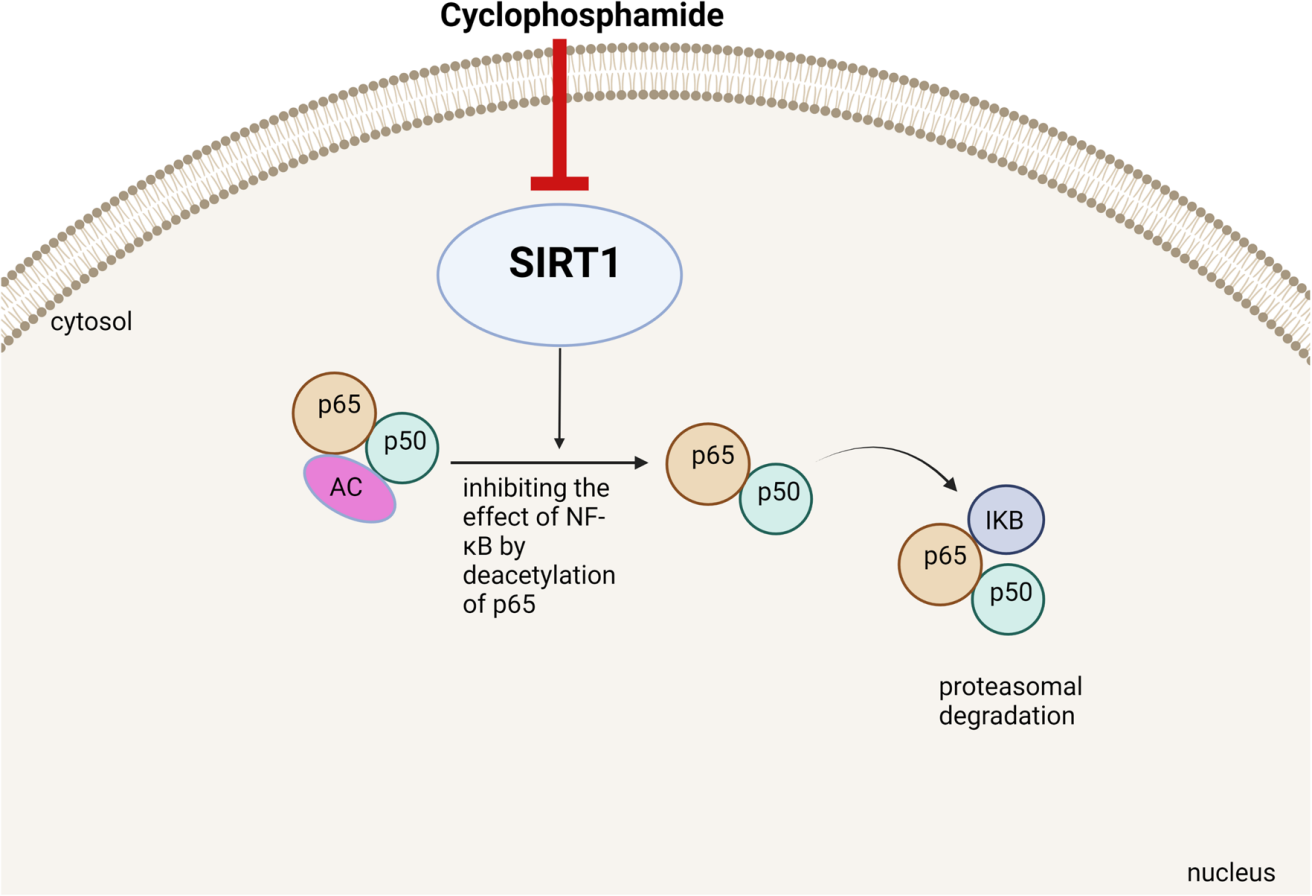
Effect of cyclophosphamide on Sirt1/ NFkB axis

SIRT-1 is one of the sirtuins family which is nicotinamide adenosine dinucleotide (NAD)- dependent deacetylase enzymes catalyzing the removal of acetyl groups from multiple targets including both histone and nonhistone proteins [54]. Downregulation of SIRT-1 has been implicated in the pathogenesis of numerous neurodegenerative diseases such as Alzheimer's disease and Parkinson's disease [55]. The neuroprotective role of SIRT-1 could be through modulating neuronal cell survival, and proliferation alongside its antioxidant, anti-inflammatory and anti-apoptotic effects [56]. In this regard, it has been stated that cyclophosphamide-brain injury is coupled with markedly diminished expression of SIRT-1 enzyme in neuronal cells resulting in boosted inflammatory responses. This could be justified by the inhibitory effect of SIRT-1 on numerous redox sensitive pro-inflammatory mediators such as NF-kB and NLRP3 [29]. SIRT-1 could repress the transcriptional activity of NF-κB by deacetylation of the p65 subunit leading to fostering the association of NF-kB complex with IkB, and thus prompting the transfer of NF-kB complex from nucleus back to the cytoplasm and diminishing the expression of the pro-inflammatory target genes [57]. Figure 6 illustrated the effect of cyclophosphamide on Sirt1/NF-kB axis.

### Induction of apoptosis

Several studies have asserted the role of apoptosis in the pathogenesis of cyclophosphamide-induced neurodegeneration [12, 19, 44]. Apoptosis could be initiated through two pathways; the extrinsic pathway, that is known as the death receptors cascade, and the intrinsic pathway, that is considered as mitochondrial-mediated event [58]. Cyclophosphamide induces apoptosis through the intrinsic pathway through generation of ROS triggering a cluster of events ultimately resulting in mitochondrial outer membrane permeabilization (MOMP) [59]. The intrinsic pathway is firmly regulated by Bcl-2 family members which includes pro-apoptotic and proteins such as Bax and Bak as well as anti-apoptotic proteins such as BCL-2 [60]. The expression of BCL-2 genes is primarily governed by the transcription factor p53 which when activated induces upregulation of the expression of the pro-apoptotic members [61].

**Fig. 7:**
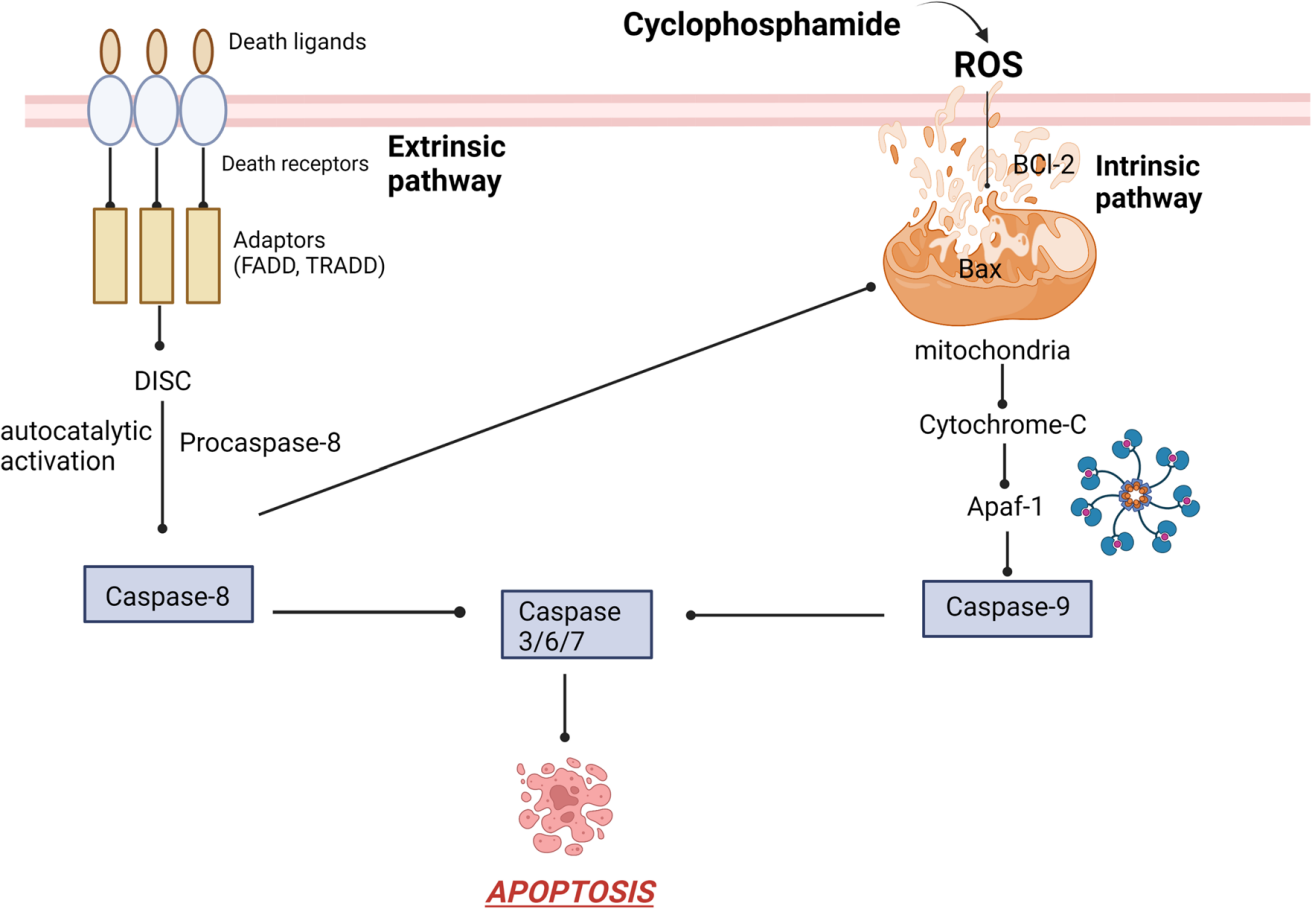
The apoptotic pathway activation in cyclophosphamide neurotoxicity. FADD: Fas-associated death domain protein, TRADD: Tumor necrosis factor receptor type 1-associated DEATH domain protein, DISC: The death-inducing signalling complex, BID: The BH3 interacting-domain death agonist, BAX: Bcl-2-associated X protein, BCL-2: B-cell lymphoma 2, Apaf-1: Apoptotic protease activating factor-1

Cyclophosphamide-induced ROS generation activate the pro-apoptotic proteins; Bax and Bak which form voids in the outer mitochondrial membrane leading to MOMP. This is followed by leakage of cytochrome c into the cytosol where it binds to pro-caspase 9 and apoptotic protease activation factor-1 (APAF-1) to form the apoptosome. This ultimately results in activation of caspase 9 which further activates the effector caspase 3 that eventually commences the apoptotic cell death. Several studies have shown that cyclophosphamide markedly elevated expression of the pro-apoptotic proteins: p53, Bax and caspase3 enzyme besides the markedly reduced expression of the anti-apoptotic protein BCL-2 [23, 25, 62, 63]. Figure 7 represents the apoptotic pathway activation in cyclophosphamide neurotoxicity.

### Effect of cyclophosphamide on neurotransmitters and neurotrophic factors

Disruption of the balance of the brain neurotransmitters and neurotrophic factors has been recently verified in diverse experimental models of chemotherapy-induced behavioral changes and cognitive deficits [64, 65]. Indeed, emerging evidence has affirmed that cyclophosphamide-induced cognitive decline is tightly coupled with cholinergic dysfunction and depletion of neuronal stores of acetylcholine (Ach) that is considered the chief neurotransmitter required for preserving normal cognition, learning and memory [15, 32]. This could be attributed to the upregulation and activation of acetylcholinesterase enzyme (AchE) which hydrolyzes Ach leading to its degradation to put an end for cholinergic neurotransmission transmission through synapses [28].

In addition, it was recently demonstrated that cyclophosphamide can also diminish brain levels of serotonin (5-HT) and dopamine further deteriorating the cognitive functions besides eliciting depression-like syndrome [28, 32]. Besides, BDNF is a vital neurotrophic factor that manipulates several cellular processes including neuronal survival, growth, proliferation, and differentiation [66]. It modulates synaptogenesis and neuronal plasticity which are essential for memory and learning [67]. Several studies have disclosed that cyclophosphamide could impair hippocampal neurogenesis by lessening BDNF expression [25, 28, 32]

## Potential protective strategies against cyclophosphamide-induced neurotoxicity

**Fig. 8:**
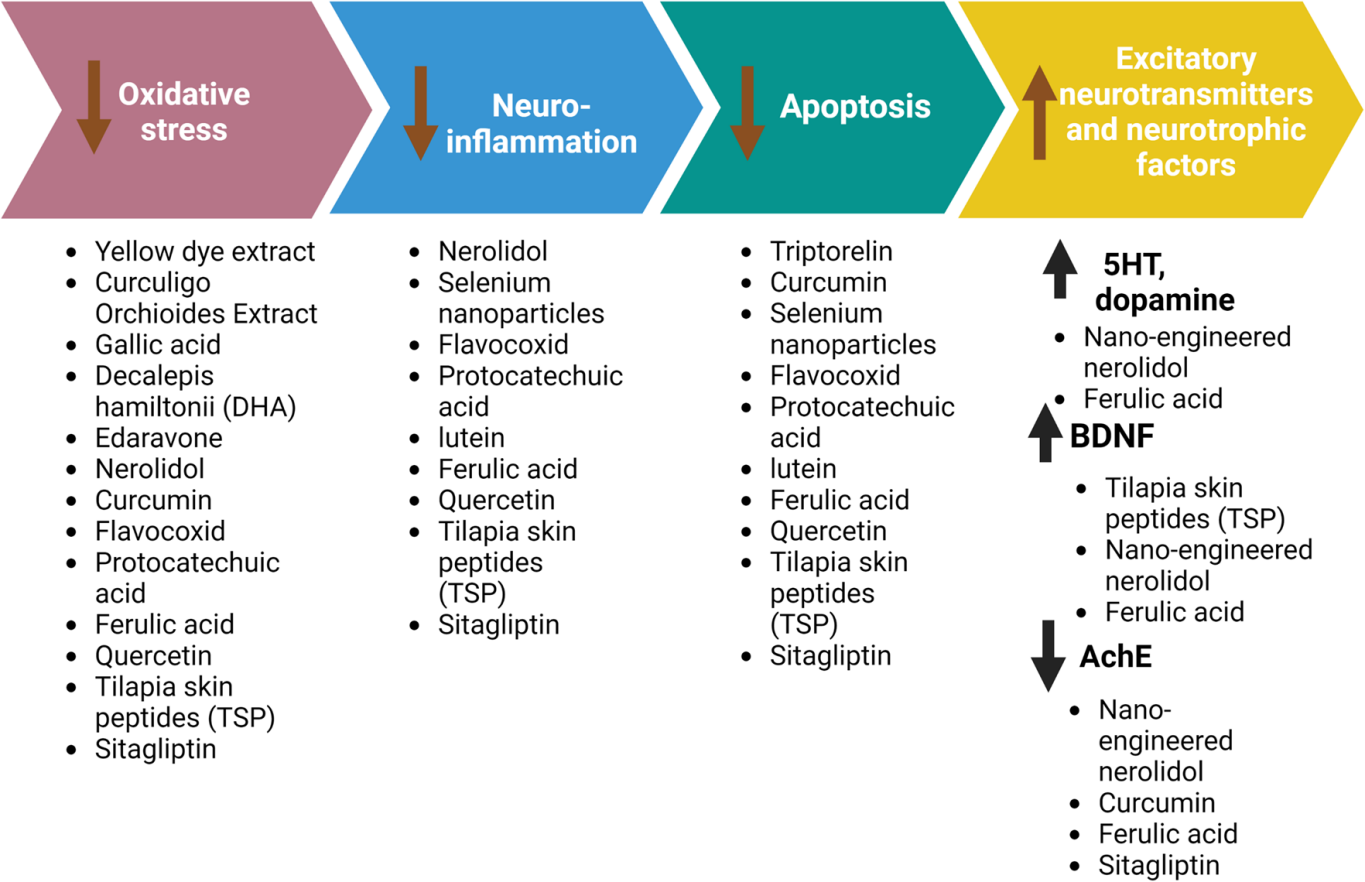
The Mechanistic targets for neuroprotective approaches against cyclophosphamide-induced neurotoxicity

Recently, cyclophosphamide-induced neurotoxicity has greatly attracted much attention for researchers. Multiple studies have been conducted to search for neuroprotective candidates to hamper this devastating complication. Based on the elucidation of the possible molecular mechanisms that may be implicated in the pathophysiology of cyclophosphamide-induced neurotoxicity, several approaches have been proposed for guarding against this threatening neurological disorder. This can be achieved through targeting oxidative stress, inflammation and apoptosis which are considered the key pathogenic mechanisms of cyclophosphamide-induced neuronal complications. Each of these preventive measures with the molecular mechanisms for their neuroprotective is detailed in Table 1 and Fig. 8.

**Table 1:** The mechanistic targets for neuroprotection against cyclophosphamide-induced neurotoxicity

Agent used	Methodology	Mechanisms of neuroprotection	References
YellowDye extract *(Morinda Lucida roots)*	Animals: Wistar albino rats Experimental design: Yellow dye 0.5% and 1% was given orally for two weeks followed by administration of cyclophosphamide (75 mg/kg, i.p.) one day before termination	Antioxidant: **↓** MDA	[69]
Curculigo Orchioides extract	Animals: Mice Experimental design: Cyclophosphamide (50 mg/kg, i.p.) was given on the first day followed by oral administration of *Curculigo Orchioides* extract at doses (200 mg/kg, and 400 mg/kg) for 5 consecutive days	Antioxidant: **↑** SOD, CAT & GPx **↓** MDA	[16]
Gallic acid	Animals: Sprague–Dawley rats Experimental design: Cyclophosphamide (100 mg/kg, i.p.) was administered on day followed by gallic acid at doses (60 mg/kg and 120 mg/kg, orally) for 10 consecutive days	Antioxidant: **↑** SOD, CAT, GPx & GST **↓** MDA, hydrogen peroxide & nitrite	[74]
Triptorelin	Animals: Male albino rats Experimental design: Cyclophosphamide (65 mg/kg/day, i.p.) was given in parallel with administration of triptorelin at doses (0.05 mg/kg/day, s.c.) for 4 weeks	Anti-apoptotic: **↓** BCL-2 **↓** p53	[62]
Decalepis hamiltonii (DHA)	Animals: Swiss albino mice Experimental design: DHA was given at doses of 50 and 100 mg/kg/day orally followed by cyclophosphamide at a doses of 25 mg/kg/day i.p. for 10 consecutive days, 1 h post DHA injection	Antioxidant: **↑** SOD, CAT, GST, GPx & GSH **↓** MDA & ROS	[13]
Edaravone	Animals: Sprague–Dawley rats Experimental design: Cyclophosphamide (100 mg/kg i.p) was given once a week along with edaravone at dose (10 mg/kg/day, orally) for one month Behavioral tests: Passive avoidance test Rotarod test	Antioxidant: **↑** SOD, CAT& GPx **↓** MDA	[14]
Nerolidol	Animals: Swiss albino mice Experimental design: *- *In silico study to assess the binding affinity of nerolidol to Nrf2 using Schrödinger software was carried out In vivo, a study was carried out using nerolidol at doses of 200 and 400 mg/kg orally followed by cyclophosphamide (200 mg/kg i.p.) on the seventh day for 2 weeks - Behavioral tests Spontaneous body alternation test, Passive avoidance test Forced swimming test	Antioxidant: **↑** Nrf2 **↑** SOD, CAT & GSH **↓** MDA Anti-inflammatory: **↓** NF-κB, TNF-α, IL-6 & IL-1β **↑** IL-10	[24]
Nano-engineered nerolidol	Animals: Swiss albino mice Experimental design: Nano-nerolidol was administered at a dose of 200 mg/kg orally for 14 days and cyclophosphamide at a dose of 200 mg/kg i.p as a single dose on day 7 Behavioral tests Morris water maze test Tail suspension test Elevated plus maze test	Antioxidant: **↓** MDA SOD, CAT & GSH Anti-inflammatory: **↓** NLPR3, caspase 1, TNF-α, IL-1β & IL-6 **↑** IL-10 Effect on neurotransmitters and neurotrophic factors: **↑** Dopamine, 5HT, BDNF **↓** AchE	[32]
Metformin	Animals: Mice Experimental design: Cyclophosphamide was administrated at doses of 100 mg/kg i.p. on alternative days for a total four doses and metformin was given at a dose of 5 mg/ml concentration dissolved in drinking water - Behavioral tests: Y-maze test Novel object recognition Test Elevated plus maze tests	–	[92]
Curcumin	Animals*:* Wistar rats Experimental design: *-*Cyclophosphamide (150 mg/kg, i.p.) a*s* single dose was given on the first day followed by curcumin at dose (20 mg/kg/day, orally) for two weeks Behavioral tests: Y-maze test Morris water maze test	Antioxidant: **↑** Thiol & non thiols proteins **↓** MDA, ACE & arginase enzyme Anti-apoptotic: **↓** Caspase-3 Effect on neurotransmitters: **↓** AchE, Butylcholinesterase Enhancing purinergic signaling: **↑** ADP & ATP	[15]
Selenium nanoparticles	Animals: Rats Experimental design: Selenium nanoparticles were given at a dose of 0.5 mg orally followed by cyclophosphamide at a dose of 20 mg/kg i.p, daily for a month	Anti-inflammatory: **↓** GFAP Anti-apoptotic: **↓** Caspase-3	[63]
Flavocoxid	Animals: Swiss albino mice Experimental design: Flavocoxid was given orally at doses (20 mg/kg) for 14 days, while cyclophosphamide was given at a dose (100 mg/kg, i.p.) on day 14	Antioxidant: **↓** MDA & APP **↑** SOD & GSH Anti-inflammatory: **↓** NF-κB, NO, TNF-α, IL-1β & GM-CSF Anti-apoptotic: **↓** Caspase-3	[17]
Protocatechuic acid	Animals: Wistar rats Experimental design: Cyclophosphamide was injected only once at a dose of 200 mg/kg, i.p. while protocatechuic acid was given at doses of 50 and 100 mg/kg orally for 10 days after cyclophosphamide injection Behavioral tests: Y-maze test Locomotor activity	Antioxidant: **↓** MDA **↑** GSH Anti-inflammatory: **↓** iNOS, galectin 3 IL-1β, IKK, NF-κB & NLRP3 **↑** SIRT1 Anti-apoptotic: **↓** Caspase-3	[29]
Lutein	Animals: Wister rats Experimental design: Cyclophosphamide was administrated as a single dose of 200 mg/kg, i.p. followed by lutein at doses; 50 and 100 mg/kg/day for 10 days	Anti-inflammatory: **↓** MIP2, CINC, MMP1, IL-1β, IL-18, NLRP3 & caspase 1	[30]
Ferulic acid	Animals: Swiss albino mice Experimental design: - Cyclophosphamide at a single dose of 200 mg/kg. i.p was given on the seventh day. While ferulic acid was given orally at two doses of 50 and 100 mg/kg/day for 2 weeks Behavioral tests: - Tail suspension test - Morris water maze test	Antioxidant: **↓** MDA **↑** SOD, CAT & GSH Anti-inflammatory: **↓** TNF-α, IL-1β & IL-6 **↑** IL-10 Effect on neurotranmitters & neurotrophic factors: **↑** Dopamine, 5-HT & BDNF **↓** AChE	[28]
Quercetin	Animals: Wister rats Experimental design: - Quercetin was given at doses of 50 mg/kg orally and cyclophosphamide at doses of 100 mg/kg dissolved in the drinking water every other day for a week - Behavioral tests Tail suspension test Open field test Y-maze tests	Antioxidant: **↓** MDA **↑** GSH, SOD, CAT, GPx & GST Anti-inflammatory: **↓** IFN-γ, IL-6, MPO, NO, IDO, TDO	[31]
Tilapia skin peptides “TSP”	Animals: C57BL/6 mice Experimental design: TSP was given at doses of 250, 500, or 1000 mg/kg/day, orally for a month and cyclophosphamide at a dose of 10 mg/kg/day, i.p. for the first 2 weeks Behavioral tests: Sucrose preference test Forced Swimming test Tail Suspension test Open Field test	Antioxidant: **↓** MDA & Keap-1 **↑** SOD, GPx, Nrf2 & HO-1 Anti-inflammatory: **↓**TNF-α, IL-1β, Iba-1 Anti-apoptotic: **↑** BCL-2 **↓** Bax & caspase-3 Effect on hippocampal Neurogenesis: **↑** DCX, BDNF & pCREB	[118]
Sitagliptin	Animals: Wistar rats Experimental design: -Sitagliptin at dose 20 mg/kg/day was given orally for 5 days before cyclophosphamide injection at a dose of 200 mg/kg, i.p. as a single dose on day 5	Antioxidant: **↓** MDA, **↑** GSH, SOD, CAT, GPx & Nrf2 Anti-inflammatory: **↓** TNF-α, IL-1β, IL-6, iNOS, NO & NF-κB Anti-apoptotic: **↓** Bax & caspase-3 Effect on neurotransmitters and neurotrophic factors: **↓** AchE	[23]

### Yellow dye extract from root of brimstone tree (*Morinda lucida*)

It is commonly utilized to enhance food quality and usually used in folk medicine. It exhibits antidiabetic, antifungal and antimalarial effect [68]. Yellow dye extract was investigated for its prominent role to protect against neurodegeneration induced by cyclophosphamide. Wistar rats received 0.5 or 1% yellow dye of *Morinda lucida* dietary plan for two weeks orally followed by i.p injection of cyclophosphamide (75 mg/kg) one day before termination. It was found that yellow dye markedly diminished the concentration of malondialdehyde (MDA), the lipid peroxidation product in rats intoxicated with cyclophosphamide. However, this reduction is noted to be in a dose-dependent manner as lipid peroxidation reduction was reported to be higher in 1% yellow dye than that of 0.5% yellow dye. This antioxidant effect could be attributed to the high phenolic concentration of *Morinda lucida*, which boosts its antioxidant capacity [69].

### *Curculigo orchioides* extract (family amaryllidaceae)

It is one of Chinese folk medicine commonly termed "Kali Musli". *Curculigo Orchioides* extract was evidenced to have immunomodulatory, anti-osteoporotic, antioxidant and anticancer properties [70]. Curculigo Orchioides extract was suggested as a promising candidate for mitigating neurotoxicity induced by cyclophosphamide [24]. In this study, mice received 50 mg/kg cyclophosphamide intraperitoneally on the first day followed by 5 days of *Curculigo Orchioides extract* administration at two doses; 200 and 400 mg/kg orally. *Curculigo Orchioides* extract could effectively guard against cyclophosphamide-indued oxidative brain damage by upgrading activities of the antioxidant enzymes; CAT, SOD, glutathione peroxidase (GPx) together with reducing brain MDA levels. These promising antioxidant capabilities of *Curculigo Orchioides extract* were attributed to the abundant content of polyphenolic compounds [71].

### Gallic acid

It is one of the well-known polyphenolic compounds found in various plants, fruits and vegetables [72]. Interestingly, gallic acid is utilized for its antiviral, antifungal, antitumor, and antioxidant activities [73]. Galic acid exhibited apparent neuroprotective properties against cyclophosphamide-induced brain damage through suppression of oxidative stress and enhancement of the antioxidant defense mechanisms. In this experimental model, rats were given a single dose of cyclophosphamide (100 mg/kg, i.p) on the first day followed by administration of gallic acid at doses (60 mg/kg and 120 mg/kg) orally for 10 days. Gallic acid successfully restored the oxidative balance by elevating antioxidant enzymes; glutathione-S-transferase (GST), GPx, CAT, and SOD, along with markedly diminishing MDA, nitrite and hydrogen peroxide levels in both cerebellar and cerebral tissues. All these findings confirmed the neuroprotective impact of gallic acid against cyclophosphamide-induced neurotoxicity [74].

### Triptorelin

It is an agonist for gonadotropin-releasing hormone [75] and considered as a cornerstone in treatment of prostate and breast cancer [76, 77]. An experimental model provided evidence for the neuroprotective influences of triptorelin against cyclophosphamide-induced neurotoxicity where rats were given cyclophosphamide (65 mg/kg/day, i.p) in conjunction with triptorelin (0.05 mg/kg/day,) subcutaneously for a month. Triptorelin significantly counteracted neurodegeneration and apoptotic aberrations caused by cyclophosphamide in both the cerebral cortex and hippocampus after one month. This was verified by the intense anti-Bcl2 staining using immunohistochemistry technique, which revealed augmented anti-apoptotic activity, while weak p53 immunostaining, which revealed decreased apoptotic activity [62].

### *Decalepis hamiltonii* (DHA) herb

This herb is grown in south India and its root has been widely consumed as pickles and juice for its high nutritional value [78]. It has been shown that DHA exerts notable antioxidant activity [79]. Besides, the roots of DHA possess neuroprotective activity [80–82]. Hence, DHA has been investigated for its protective potential against cyclophosphamide neurotoxicity where mice were given DHA orally at doses of 50 and 100 mg/kg for 10 days followed by cyclophosphamide at a dose of 25 mg/kg i.p. for 10 days after 1 h of DHA administration. DHA halted the progression of cyclophosphamide oxidative damage in brain tissues as demonstrated by reduction in ROS, lipid peroxidation levels as well as elevation of GSH levels, besides the activities of the antioxidant enzyme; SOD, CAT, GST, GPx and GR [13].

### Edaravone

It is a synthetic medication used for management of acute ischemic stroke due to its neuroprotective reagent, and superior antioxidant properties [83, 84]. It has been examined for its neuroprotection against cyclophosphamide-induced cognitive impairment and motor dysfunction. A combination of cyclophosphamide (100 mg/kg/week, i.p) and edaravone (10 mg/kg/day, orally) was given to rats for 4 weeks. Edaravone improved motor functions and long-term memory as shown by the findings of rotarod and passive avoidance tests, respectively. Besides, it regained the redox balance by rising GSH levels and activities of CAT, SOD and GPx enzymes while reducing MDA levels [14].

### Nerolidol

Nerolidol is a sesquiterpene compound having promising antioxidant and anti-inflammatory properties [85, 86]. Notably, nerolidol has exhibited worthy neuroprotective activity [87, 88]. Iqbual et al. [18] has examined the effect of nerolidol on cyclophosphamide-induced chemobrain. In this study, mice were given nerolidol orally at doses of 200 or 400 mg/kg for 14 days then cyclophosphamide (200 mg/kg i.p.) was given on the seventh day. Nerolidol could efficiently improve the cognition functions as shown by results of the behavioral tests; spontaneous body alternation and passive avoidance test. Additionally, it could alleviate cyclophosphamide-induced depression as demonstrated by the findings of the forced swimming test. Mechanistically, nerolidol remarkably preserved redox balance in both hippocampal and cortical tissues by elevating GSH levels, CAT and SOD activities while reducing MDA levels. In addition, it showed also anti-inflammatory effects by diminishing cortical and hippocampal content of the pro-inflammatory cytokines; TNF-α, IL-6 and IL-1β while rising the content of the anti-inflammatory cytokine; IL-10. Immunohistochemical analysis revealed that nerolidol-induced upregulation of Nrf2 and downregulation of the NF-κB which accounts for its antioxidant and anti-inflammatory effects, respectively. Moreover, an *in silco* study was done to confirm the binding affinity of nerolidol to Nrf2 using Schrödinger software [24]

The neuroprotective capabilities of nerolidol against cyclophosphamide neurotoxicity were further affirmed by *Iqbual *et al., in a novel delivery system in which nano-engineered loaded lipid carrier was used to enhance BBB penetration. Notably, it was reported that nerolidol nano formulation could alleviate cyclophosphamide-induced depression as evident from the results of the tail suspension test. This has been linked to uprising levels of dopamine and 5-HT in both the hippocampus and cortex. In addition, the findings of Morris water maze and elevated plus maze tests further supported the positive impact of nerolidol nano formulation on cognitive function which was coupled with inhibiting activity of AchE. Besides, nerolidol nano formulation has markedly elevated the levels of BDNF augmenting neuronal survival and proliferation. Additionally, this study has provided further evidence for the antioxidant and anti-inflammatory capabilities of nerolidol. Moreover, additional molecular mechanism has been proposed for the anti-inflammatory effects of nerolidol through suppressing NLPR3 inflammasome/ caspase 1 signaling cascade. Of interest, the molecular docking studies have proved strong binding affinity of nerolidol with both NLRP3 and TLR-4 proteins. This study concluded that nerolidol in the nano formulation has better neuroprotective abilities than the conventional form at the same dose owning to the enhanced ability to cross BBB due to its higher lipophilicity and the smaller particle size [32].

### Metformin

It is a well-known antidiabetic drug that belongs to the biguanide family [89]. It exerts its antidiabetic effects by augmenting insulin receptor sensitivity [90]. A previous study showed that metformin could alleviate cognitive impairment in diabetic mice [91].In this study, mice received 100 mg/kg of cyclophosphamide i.p. on alternate days for a total of four doses and/or metformin dissolved in the mice’s drinking water at a concentration of 5 mg/ml from day zero to the termination of the treatment cycles. Cognitive functions were evaluated using behavioral examination techniques such as Y-maze, novel object recognition, and elevated plus maze tests. The findings of these behavioral tests manifested that metformin could ameliorate cognitive dysfunction coupled with cyclophosphamide [92].

### Curcumin

It is a polyphenolic compound obtained from turmeric rhizomes [93].Its wide usage arises from its minimal side effects alongside its plentiful therapeutic benefits such as antioxidant, anti-inflammatory, anticancer, antimicrobial and antidiabetic effects [94–97]Curcumin has been shown to have eminent neuroprotective properties in a variety of neurodegenerative diseases, including Alzheimer disease and Parkinson disease [98]. Likewise, *Seun Funmilola Akomolafe *et al. confirmed the effect of curcumin in prevention of cognitive impairment caused by cyclophosphamide. Rats were given cyclophosphamide (150 mg/kg, i.p.) on day 1 of the study and curcumin (20 mg/kg/day, orally) for 14 days. The results of water maze and Y-maze behavioral tests disclosed that curcumin improved rats' cognitive abilities. Besides, it could preserve cholinergic neurotransmission due to inhibition of AchE and butyrylcholinesterase. Curcumin was also found to improve redox equilibrium as manifested by decreasing MDA levels, while increasing thiol levels. These antioxidant effects were linked with reducing activities of arginase and ACE enzymes that triggers ROS production. In addition, it enhanced the purinergic signaling pathway presented by the increase in ATP and ADP brain levels. Furthermore, immunohistochemical analysis showed reduced cleaved caspase-3 enzyme immunoreactivity upon curcumin administration implying its anti-apoptotic effects [15].

### Selenium

It is a trace element with a variety of health benefits. It is one of the most extensively investigated micronutrient for prevention of several diseases by the virtue of being antioxidant and cytoprotective agent [99]. Selenium is proved to possess a broad spectrum of biological functions, including antioxidant, anti-inflammatory, as well as antitumor effects. [100–102], Selenium nanoparticles were studied to protect against cyclophosphamide-induced neurodegeneration [63]. Rats were give selenium nanoparticles at dose of 0.5 mg/kg, followed by cyclophosphamide (20 mg/kg,i.p.) on daily basis for a month. Immunohistochemical analyses of the hippocampal expression of caspase 3 enzyme and glial fibrillary acidic protein (GFAP), a marker for astrocytic activity, were assessed. Selenium nanoparticles provoked anti-inflammatory and anti-apoptotic activities evidenced by preserving normal levels of GFAP and caspase 3 proteins [63].

### Flavocoxid

It is a medicinal food composed of a mixture of the flavonoids; baicalin, and catechin. It has been frequently utilized for management of various inflammatory disorders because of its potent anti-inflammatory activity mediated by blunting activity of the pro-inflammatory enzymes including cyclo-oxygenase and lipoxygenases [103]. Besides, it has been confirmed that flavocoxid has superior anti-oxidant abilities [104, 105]. The neuroprotective impact of flavocoxid against the neurotoxicity of cyclophosphamide was investigated [30]. Mice were given flavocoxid at a dose of 20 mg/kg orally for 14 days, and a single dose (100 mg/kg, i.p) of cyclophosphamide on day 14. The findings demonstrated that flavocoxid exerts antioxidant effects as it markedly reduced MDA and elevated GSH and SOD brain concentrations. These antioxidant effects of flavocoxid were coupled with diminishing levels of brain amyloid precursor protein (APP) assessed by western blot analysis. In addition, flavocoxid showed anti-inflammatory properties by decreasing levels of NO, granulocyte macrophage colony stimulator factor (GM-CSF), TNF-α, and IL-1β which could be explained by downregulating expression of NF-κB measured by western blot technique. Moreover, caspase-3 immunostaining showed reduced enzyme expression following flavocoxid treatment denoting its anti-apoptotic effects [17].

### Protocatechuic acid

It is a polyphenolic compound present in numerous plants such as green and black tea [106]. It has diverse pharmacological activities involving anti-inflammatory, antioxidant, antibacterial, antiviral, anticancer and antidiabetic [107]. Growing evidence has revealed the therapeutic benefits of protocatechuic acid in a diversity of neurodegenerative diseases [108]. In this context, the neuroprotective impact of protocatechuic acid on chemobrain caused by cyclophosphamide has been recently evaluated [29]. Rats were given cyclophosphamide once at a dose of 200 mg/kg, while protocatechuic acid was given at a dose of 50 and 100 mg/kg orally for 10 days after cyclophosphamide injection. The results of Y-maze test and locomotor activity assessment proved the positive impact of protocatechuic acid on cognitive abilities and motor function. Protocatechuic acid showed neuroprotection through its antioxidant effects by reducing MDA and increasing GSH levels. Besides, it markedly diminished gene expression of inducible nitric oxide synthase (iNOS) assessed by real time PCR technique, as well as protein levels of IL-1β, IKK, NF-κB, NLRP3 and galectin 3 emphasizing its potent anti-inflammatory effects. Additionally, its neuroprotective effects were associated with upregulating expression of SIRT1 that has considerable anti-inflammatory roles. Further, its anti-apoptotic activity was demonstrated by decreasing caspase 3 gene expression examined using real time PCR technique [29].

### Lutein

It is a carotenoid compound produced by plants, bacteria, and microalgae [109]. Accumulating evidence has confirmed its anti-oxidant, and anti-inflammatory properties [110]. It is distinguished by its valuable health benefits in multiple pathological disorders such as ocular complications, cardiovascular diseases, microbial infections, and neurological disorders [111]. The modulatory effect of lutein on the neurotoxicity of cyclophosphamide was recently studies. Rats were given cyclophosphamide as a single dose of 200 mg/kg, i.p. followed by lutein at doses 50 and 100 mg/kg for 10 days. Lutein significantly counteracted cyclophosphamide-induced neuroinflammation as shown by lowering the brain levels of the pro-inflammatory mediators; macrophage inflammatory protein2 (MIP2), cytokine-induced- neutrophil chemoattractant (CINC), a matrix metalloproteinase 1 (MMP1), IL-1β, IL-18, NLRP3 and caspase 1 [30].

### Ferulic acid

It is a naturally occurring hydroxycinnamic derivative that is widely distributed in a multiplicity of fruits and vegetables [112]. It is familiar with its compelling free radical scavenging activity with multiple commercial utilities particularly in cosmetic and pharmaceutical industries [113]. Besides, it has been reported for its anti-inflammatory, anti-thrombotic, anticancer, antihyperlipidemic, antimicrobial activity and antidiabetic effect [114]. *Mishra *et al*.,* illustrated the advantageous influence of ferulic acid on behavior and cognition in an experimental murine model of cyclophosphamide-induced neurotoxicity. Cyclophosphamide at a single dose of 200 mg/kg. i.p was given on the 7^th^ day while ferulic acid was given orally at two doses of 50 and 100 mg/kg for 2 weeks. The findings of the tail suspension test indicated that ferulic acid could alleviate cyclophosphamide- induced depression which was explained by elevating levels of the excitatory neurotransmitters; 5-HT and dopamine. Besides, Morris Water Maze results also affirmed positive effects of ferulic acid on cognitive function which was attributed to enhancement of cholinergic neurotransmission through inhibiting activity of AchE. Moreover, ferulic acid could also enhance neurogenesis as shown by the elevated levels of the neurotrophic factor; BDNF. The results also showed that ferulic acid significantly elevated GSH level and activities of CAT and SOD enzymes in hippocampal and cortical tissues while hindered lipid peroxidation. Beside repressing cyclophosphamide-induced oxidative injury, ferulic acid also prohibited the inflammatory reactions by decreasing levels of the inflammatory cytokines; IL-1β, IL-6 and TNF-α along with rising the levels of IL-10 [28].

### Quercetin

It is a polyphenolic flavonoid found in low quantities as a secondary metabolic product in various fruits and vegetables [115]. It is marketed as a nutritional supplement with a wide range of biological activities such as, anti-inflammatory, antioxidant, anti-allergic, antihypertensive, antihyperlipidemic and neuroprotective [116]. It was investigated by *Ebokaiwe *et al*.,* for its role in attenuating cyclophosphamide-induced cortical and hippocampal neurodegeneration. Rats were treated with quercetin at a dose of 50 mg/kg orally and cyclophosphamide at a dose of 100 mg/kg in the drinking water every other day for a week. Several behavioral tests have been conducted such as tail suspension, open field, and Y-maze tests implying the relieving effects of quercetin on cyclophosphamide-indued depressive like behavior and cognitive decline. Concerning the molecular mechanism of these promising neuroprotective effects, quercetin induced a marked elevation in GSH level and activities of antioxidant enzymes; SOD, CAT, GPx and GST while reduced MDA level. Moreover, it efficiently halted the inflammatory responses by diminishing levels of pro-inflammatory mediators including IL-6, IFN-γ, NO and activity of myeloperoxidase enzyme (MPO). The anti-inflammatory effects of quercetin were accompanied by its inhibitory effects on the enzymes; indoleamine 2,3‐dioxygenase (IDO1 and tryptophan 2,3‐dioxygenase (TDO). Moreover, western blot and immunohistochemistry techniques further showed downregulation of IDO1 enzyme expression following quercetin administration. Indeed, both IDO and TDO enzymes responsible for tryptophan degradation subsequently leading to eliciting local inflammation signals in brain tissues [31].

### Tilapia skin peptides “TSP”

They are blend of low molecular-weight marine peptides originated from tilapia skin during fish processing [117]. TSP have been found to possess several biological activities such as antioxidant, anti-inflammatory, anti-apoptotic effect and wound healing [118]. Zhao et al., studied the effect of TSP on anxiety and depression-like behavior associated with cyclophosphamide intoxication in mice. TSP was given orally at doses; 250, 500, and 1000 mg/kg/day for a month while cyclophosphamide was given intraperitoneally at dose of 10 mg/kg/day for the first two weeks of the experiment. Multiple behavioral tests demonstrated the remedial effects of TSP effects on depression and anxiety as evidenced by uprise in the preference of sugar water, shortening the immobility time during both the forced swimming test and tail suspension test besides increasing traveling distance within the open field test. TSP at dose 1000 mg/kg/day almost normalized the hippocampal MDA level beside SOD and GPx enzyme activities signifying its potent antioxidant impact. The upregulation of antioxidant enzymes can be mediated by stimulating Nrf2 signaling cascade as manifested by markedly decreased expression of Keap-1 protein alongside the substantially elevated expression of Nrf2 and, HO-1 proteins which were all examined by western blot technique. Moreover, TSP suppressed neuroinflammation in the hippocampus through reducing levels of the inflammatory cytokines; TNF-α, and IL-1β along with ionized calcium-binding adapter molecule 1 (Iba-1); a marker for microglial activation as imvestigated by immunofluorescence. In addition, the reduced percent of positively stained cells in tunnel assay for apoptotic neurons proved the anti-apoptotic effects of TSP which was linked with upgrading Bcl-2 expression while downgrading Bax and caspase-3 expressions as evidenced by the results of western blot analysis of these proteins. In addition, the beneficial effect of TSP on hippocampal neurogenesis was evident from the increased expression of the neurogenesis marker; doublecortin (DCX) as shown by the elevated number of DCX positive cells in the immunofluorescence assay. This aligns with TSP-induced elevated expression of BDNF consequently activating its downstream signaling pathway as manifested by the raised expression of the active phosphorylated form of the transcription factor; cAMP response element-binding protein (CREB) which plays pivotal roles in conserving adult hippocampal neurogenesis. The BDNF/CREB signaling cascade was investigated using western blot assay [118].

### Sitagliptin

It is an oral hypoglycemic drug, exerts its antidiabetic effects by inhibiting the dipeptidyl peptidase 4 (DPP-4) enzyme, thus repressing incretin degradation and stimulating insulin secretion [116]. Supporting evidence has asserted the promising neuroprotective potential of sitagliptin and its valuable impact on hippocampal neurogenesis and cognitive function [119–121]*. Famurewa *et al*.* has investigated the effectiveness of sitagliptin in mitigating cyclophosphamide-induced cerebral neurotoxicity in an experimental rat model. Sitagliptin (20 mg/kg/day, orally) was given for 5 days before cyclophosphamide administration at a single dose of 200 mg/kg, i.p on day 5. Sitagliptin exhibited antioxidant effects mediated by elevating Nrf2 expression subsequently leading to upregulating cerebral antioxidant defenses such as GSH, SOD, CAT and GPx and prohibiting lipid peroxidation. Besides, it demonstrated potent anti-inflammatory effects confirmed by diminishing the levels of the pro-inflammatory markers; NO, iNOS, IL-1β, IL-6, and TNF-α through reducing NF-κB expression. Moreover, the anti-apoptotic effect of sitagliptin manifested by lessening expression of Bax and caspase 3 enzyme further contributed to protection against cyclophosphamide-induced cerebral neuronal damage. Finally, it could restore normal cognitive function through heightening cholinergic conduction by inhibiting AchE [23].

## Limitations

A major limitation of the current review is the paucity of the clinical reports documenting the neurological complications associated with cyclophosphamide usage in cancer patients. Besides, another critical limitation is the lack of clinical trials for evaluating the potential role of the promising neuroprotective candidates investigated in the preclinical studies for alleviating cyclophosphamide-induced neurotoxicity in cancer patients. Furthermore, the effects of these potential candidates on the antitumor activity of cyclophosphamide haves not been yet evaluated in either in vivo experiments or clinical trials.

## Future prospective

Cyclophosphamide is widely used as chemotherapeutic agent in treatment of various types of human malignancies. However, its clinical utility is considerably hampered due to multiple toxicities including neurotoxicity. Several in vivo experiments have been conducted for investigating the molecular mechanisms underlying this debilitating neurotoxicity. However, more studies are still warranted for further exploration of other mechanistic molecular targets that may play role in the pathophysiology of cyclophosphamide-induced neurotoxicity such as involvement of protein oxidation and mitochondrial DNA damage in mediating this adverse effect. Of note, multiple preclinical studies have been carried out for evaluating potential neuroprotective effects of many synthetic drugs and natural phytochemicals against this neurological complication. However, there is a lack of clinical evidence for the therapeutic efficacy of these candidates in alleviating neurological complications in cancer patients treated with cyclophosphamide. Therefore, well-designed randomized clinical trials should be conducted in order to validate the therapeutic benefits of these neuroprotective agents for cancer patients. Furthermore, the effect of these potential candidates on the cytotoxic effect of cyclophosphamide has not been yet evaluated. Thus, both preclinical and clinical studies should be performed to investigate the interaction of these neuroprotective agents with the antitumor activity of cyclophosphamide.

## Conclusions

The purpose of this review is to systematically elucidate the molecular mechanisms behind cyclophosphamide-induced neurotoxicity and the potential protective strategies for counteracting this neurological complication. The findings of several experimental studies have unveiled diverse molecular targets that could contribute to the pathogenesis of cyclophosphamide neurotoxicity. Among them, oxidative stress is considered the cornerstone where cyclophosphamide elicits massive generation of ROS coupled with downregulating activities of antioxidant enzymes alongside Nrf2 pathway. In addition, neuroinflammation has been extensively verified as a key pathological event where cyclophosphamide provokes the release of a battery of pro-inflammatory cytokines through upregulating various inflammatory signaling cascades as NFκB, TLR and NLRP3 while downregulating Sirt-1 expression. In addition, various studies haver affirmed the role of mitochondrial-dependent apoptotic cell death and modulating expression of Bcl-2 family proteins in mediating this neurotoxicity. Furthermore, it has been stated that cyclophosphamide neurotoxicity is accompanied by repressing cholinergic neurotransmission by activating AchE and disrupting the balance of the brain neurotransmitters such as dopamine and 5-HT and neurotrophic factors as BDNF. Diverse experimental studies have been conducted to search for potential neuroprotective agents either synthetic drugs or phytochemical compounds for mitigating this neurological complication beside investigating the possible mechanisms underlying this neuroprotection. However, to date, there is no clinically FDA approved agent to be used as adjuvant therapy with cyclophosphamide for ameliorating the associated neurotoxicity. Thus, it is highly recommended to carry out clinical trials to verify the usage of these candidates safely, and to validate their effects on the antitumor activity of cyclophosphamide.

## Data Availability

Enquiries about data availability should be directed to the authors.
